# 261. A Rare Case of Meningitis and Symptomatic Hydrocephalus by Listeria Monocytogenes in Dermatomyositis: A Case Report

**DOI:** 10.1093/ofid/ofab466.463

**Published:** 2021-12-04

**Authors:** Eylem Kiral, Ebru kacmaz, Gurkan Bozan, Ozgur Arslanoglu, Omer Kilic, Ener Cagri Dinleyici

**Affiliations:** 1 ESOGÜ, Eskisehr, Eskisehir, Turkey; 2 ESOGU, Eskisehir, Eskisehir, Turkey

## Abstract

**Background:**

Listeria monocytogenes is a gram-positive, facultative anaerobic bacillus common in the intestinal flora of many animals and humans. We describe an unusual case of meningitis by Listeria monocytogenes (LM) complicated by hydrocephalus in a child with dermatomyositis.

**Methods:**

A 15-year-old girl presented to an outside hospital (OH) after a three-day history of headache, fever and was hospitalized with a diagnosis of meningitis and lumbar puncture performed. CSF sample could not be evaluated clearly due to its hemorrhagic nature. Her past medical history was significant for dermatomyositis for five years. She had received induction of IVIG five days prior. She was also taking cyclosporin A and hydroxychloroquine. She was empirically treated with intravenous cefotaxime, vancomycin, and acyclovir. She was urgently transferred to the theatre for an external shunt placement in the right lateral ventricle. The interval between the first symptoms and the diagnosis of hydrocephalus was around 4 days. CSF from this catheter showed growth of LM with sensitivity to meropenem and resistance to erythromycin, ampicillin, and sulfamethoxazole-trimethoprim. Gram staining of CSF resulted negative for bacteria. Cefotaxime was switched to intravenous meropenem. Immunological screening of cellular and humoral immunity, complement, and blood iron levels were normal. SARS-Cov2 PCR and HIV tests were negative. Herpes virus, mycobacterium tuberculosis real-time PCR, respiratory viral panel studied in the CSF sample were negative. MRI and Angio of the brain showed no abnormality. She is being followed in the pediatric intensive care unit as intubated.

**Results:**

In patients who received immunosuppressive medication, L. monocytogenes should be evaluated in the differential diagnosis of central nervous system infections. Even if effective antibiotic therapy has been initiated, this case highlights the need of recognizing early hydrocephalus as a consequence of Listeria meningitis in children with neurological deterioration a few days after initial presentation.

**Conclusion:**

The literature on the management and outcome of Listeria meningitis-related hydrocephalus in children is limited.

**Disclosures:**

**All Authors**: No reported disclosures

